# Targeting angiogenesis in Duchenne muscular dystrophy

**DOI:** 10.1007/s00018-019-03006-7

**Published:** 2019-02-15

**Authors:** Paulina Podkalicka, Olga Mucha, Jozef Dulak, Agnieszka Loboda

**Affiliations:** Department of Medical Biotechnology, Faculty of Biochemistry, Biophysics and Biotechnology, Jagiellonian University, Gronostajowa 7, 30-387 Kraków, Poland

**Keywords:** Dystrophy, Angiogenesis, *mdx*, Heme oxygenase-1, Statins

## Abstract

Duchenne muscular dystrophy (DMD) represents one of the most devastating types of muscular dystrophies which affect boys already at early childhood. Despite the fact that the primary cause of the disease, namely the lack of functional dystrophin is known already for more than 30 years, DMD still remains an incurable disease. Thus, an enormous effort has been made during recent years to reveal novel mechanisms that could provide therapeutic targets for DMD, especially because glucocorticoids treatment acts mostly symptomatic and exerts many side effects, whereas the effectiveness of genetic approaches aiming at the restoration of functional dystrophin is under the constant debate. Taking into account that dystrophin expression is not restricted to muscle cells, but is present also in, e.g., endothelial cells, alterations in angiogenesis process have been proposed to have a significant impact on DMD progression. Indeed, already before the discovery of dystrophin, several abnormalities in blood vessels structure and function have been revealed, suggesting that targeting angiogenesis could be beneficial in DMD. In this review, we will summarize current knowledge about the angiogenesis status both in animal models of DMD as well as in DMD patients, focusing on different organs as well as age- and sex-dependent effects. Moreover, we will critically discuss some approaches such as modulation of vascular endothelial growth factor or nitric oxide related pathways, to enhance angiogenesis and attenuate the dystrophic phenotype. Additionally, we will suggest the potential role of other mediators, such as heme oxygenase-1 or statins in those processes.

## Duchenne muscular dystrophy and other dystrophies

Duchenne muscular dystrophy (DMD, MIM #310200) is one of the most severe forms of inherited muscular dystrophies. It is a devastating X-linked genetic disorder that affects approximately 1 in 5000–6000 boys [1] and is caused by the mutation in the dystrophin gene (DMD, MIM #300377), the largest gene in the human genome, located on the chromosome Xp21 [2]. Several syndromes associated with progressive skeletal muscle wasting and degeneration diseases (known as dystrophinopathies) are described, with Becker muscular dystrophy (BMD) and limb-girdle muscular dystrophies (LGMD) being the more frequently diagnosed after DMD [3]. The *DMD* gene contains 79 exons, encodes a 14-kb mRNA and produces the protein product with a molecular weight of 427 kDa [4, 5]. The most common forms of the mutations leading to DMD are the intragenic deletions and duplications (they account for over two-thirds of the mutations) with point mutations detected in 20–30% of patients [4]. In-frame mutations causing the truncation of the protein result rather in the much milder disease, BMD [6].

Dystrophin is a crucial component of the dystrophin-associated protein complex, responsible for the connection of the sarcolemma and extracellular matrix (ECM) to the actin cytoskeleton within skeletal myofibers and cardiomyocytes [7]. The postulated role of dystrophin is to protect the sarcolemma from the stress of repeated contractions by providing an indirect link between the subsarcolemmal actin and the intermediate filaments in the muscle fiber with ECM components. Therefore, mutations in the dystrophin gene resulting in the lack of functional dystrophin cause mechanical instability and myofibers destruction with repeated cycles of necrosis and regeneration as well as inflammatory response. Degenerating myofibers accumulate immunoglobulins IgA and IgG [8] and release creatine kinase (CK) as well as lactate dehydrogenase (LDH) that can be detected in the plasma as markers of muscle damage [9, 10]. Neutrophils and pro-inflammatory macrophages invade dystrophic muscles to remove debris and by secretion of Th1 cytokines, they regulate the activation, proliferation, migration, and differentiation of satellite cells (SCs), muscle stem cells. Pro-inflammatory cytokines and membrane instability lead to self-sustaining activation of the innate immune response—induction of MHC I and II on muscle cells, recruitment of Th and Tc lymphocytes and constant damage of muscles. From the other hand, Treg cells try to compensate the pro-inflammatory effects as they secrete immunosuppressive IL-10 and reduce expression of IFNγ released by Th lymphocytes [11, 12]. In dystrophic muscles, continuous cycles of damage and inflammation over years lead to the replacement of muscles by fibrous connective tissues and fat, and as a consequence, improper and final loss of muscle function [11, 13].

Still, new processes and molecular pathways are identified to play an important role in the modulation of DMD progression. The increased oxidative stress may affect both autophagy and mitochondrial respiration. In fact, impairment in the autophagy process, leading to the accumulation of damaged organelles, was reported in muscles from DMD patients [14]. Moreover, the mitochondrial dysfunction in dystrophic skeletal muscle is well documented and it not only contributes to the reductions in resting ATP content but also leads to the impairment of autophagy, apoptosis, inflammation, fibrosis, and satellite cells death (reviewed in [15]).

Recent studies concentrate more on the involvement of SCs in DMD progression. Until recently dystrophin was thought to be expressed only in myotubes and myofibres, but its presence was also confirmed in other cell types, including muscle stem cells [16]. In SCs, dystrophin is responsible for the maintenance of the appropriate cell polarity during the cell division. As a consequence of dystrophin deficiency, dysfunction of asymmetric SCs division and cell polarity has been observed resulting in the inefficient generation of myogenic progenitors and impaired muscle regeneration [16]. This would explain the fact that although the number of SCs in *mdx* mice is increased, their regenerative capacity is compromised by the alterations in SCs self-renewal and maintenance. Moreover, it might be suggested that observed defects can be translated to DMD patients as well, emphasizing that DMD pathology, except direct muscle weakening and fragility, should be considered as the muscle stem cell disease [16]. Moreover, our group has revealed some notable alterations in the shape of differentiating SCs isolated from dystrophic mice, what could potentially reflect their functional impairment [10].

**Fig. 1:**
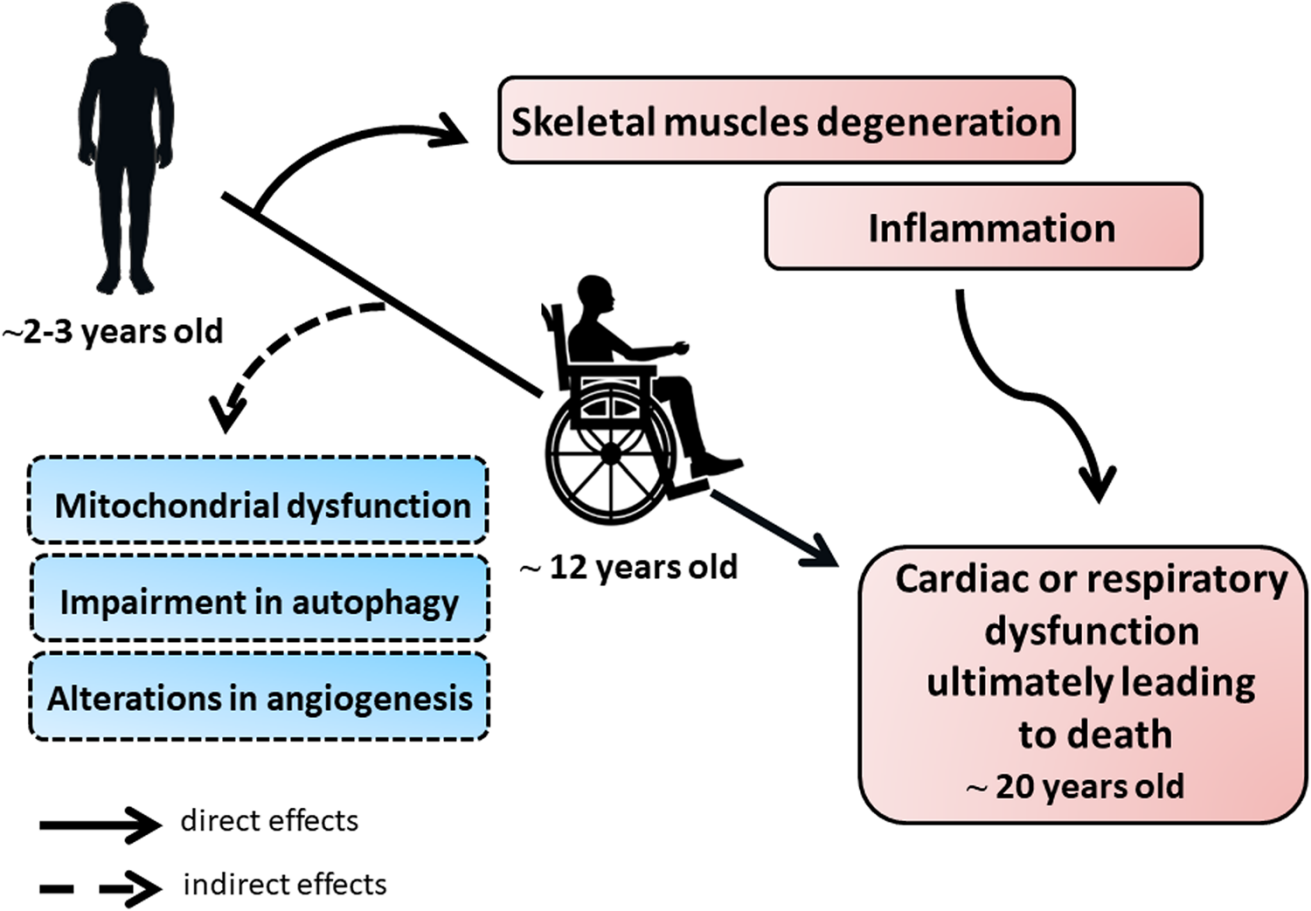
The pathology of Duchenne muscular dystrophy (DMD). The progression of DMD proceeds rapidly due to the several direct and indirect effects. First symptoms of the disease start in early childhood, around the age of 2–3, with skeletal muscles degeneration and weakness being the primary cause of dystrophin deficiency. Concomitantly, repeated cycles of necrosis and regeneration of muscle fibers trigger a strong immune response. As the consequence, patients lose the ability to walk around the age of 12 and finally, around the age of 20, they die due to the cardiac or respiratory dysfunction. As no complete cure for DMD patients exists currently, investigation of new processes and molecular pathways that could play an important role in DMD is still needed. Accordingly, mitochondrial dysfunction, as well as impairment in autophagy and possibly angiogenesis processes, was also suggested to contribute to the progression of the disease

First symptoms of the disease start in early childhood with skeletal muscle weakness, including fatigue, difficulty in standing up (the so-called Gower’s maneuver), walking as well as frequent falls and a characteristic Trendelenburg gait. Unfortunately, the disease progresses rapidly, and around the age of 12, patients lose the ability to walk, develop spinal curvature known as kyphosis, paralysis and finally die due to respiratory or cardiac failure (dilated cardiomyopathy) around 20 years of age [17] (summarized in Fig. 1).

There is no cure for patients suffering from muscular dystrophies currently. Glucocorticoids (GCs), with prednisolone and deflazacort being most commonly used, still serve as a gold standard therapy, acting mostly as anti-inflammatory drugs [18]. With the use of steroids and multidisciplinary care, particularly the mechanical ventilation, the lifespan of some affected individuals can be now extended to 30–40 years of age. Nonetheless, except clear, long-term beneficial effects of GCs [19], their daily administration is associated with various adverse effects [20]. Although Quattrocelli et al. [21] showed that weekly, intermittent GCs administration provides a better alternative to a daily regimen without eliciting muscle atrophy, still pharmacological approaches are mainly mitigating the symptoms of the disease, acting as non-specific agents. Thus, during recent years, a great amount of effort has been put into the investigation of novel, gene-based therapies, such as exon skipping approach or suppression of stop codons.

Recently, more attention is paid to induced pluripotent stem cells (iPSCs) technology and their potential application in DMD treatment. Many studies have described various attempts to develop and characterize human iPSC-based models of DMD (summarized in [22]) and iPSCs differentiated to cardiomyocytes to model dilated cardiomyopathy observed in DMD patients [23, 24]. In addition, Filareto et al. [25] using corrected-iPSC-derived myogenic precursors (derived from *mdx*/utrn^−/−^ mice fibroblasts and engineered to express a micro-utrophin, Pax3 and Pax7) showed that they are able to engraft in vivo following local and systemic transplantation and this resulted in improved muscle strength of *mdx*/utrn^−/−^ mice.

Furthermore, with the development of CRISPR/Cas9 technology the possibilities for the restoration of functional dystrophin arisen dramatically [26]. For example, Young et al. [27] have found that removal of exons 45–55 resulted in the expression of the stable dystrophin protein in both cardiomyocytes and skeletal myotubes in vitro. Such genetic manipulation caused functional effects as improved membrane integrity and CK level similar to the wild-type cells was measured in reframed cells. Nonetheless, although an increasing number of studies report successful and beneficial effects of CRISPR/Cas9 in animal models of muscular dystrophy [28–30], genome editing tools are still far from being therapeutically available. Accordingly, there is a constant need for investigation of novel, molecular targets that could provide potential treatment options for patients suffering from muscular dystrophies.

## Animal models of DMD

To better understand the basic biology behind DMD pathology, several animal models of the disease, ranging from non-mammalians such as *Caenorhabditis elegans* [31] and zebrafish [32], through the mouse and canine models have been created, and the list is still growing. As the majority of models were already thoroughly described in many review papers [33–35], we will only briefly present some of the most commonly used ones in order to illustrate the complexity of the subject.

### Mouse model—*mdx* mice

Commonly used are mice with the spontaneous, nonsense, point mutation in 23 exon of the dystrophin gene, the so-called *mdx* (C57BL/10ScSn genetic background) mice [35–37], with over 3000 publications on Pubmed to date. Similar to the DMD, *mdx* mice lack functional dystrophin protein what first suggested that this model might be the equivalent of the human disease in mice [38]. Moreover, typical histological features such as muscle degeneration, a variation of the fiber size and occurrence of centrally nucleated, regenerating fibers can be observed in these mice [36]. However, the timeline and intensity of the symptoms differ between *mdx* animals and DMD patients. In humans, muscle degeneration is progressive through the whole life of the patients, with muscle tissue being continuously replaced by fibro-fatty connective tissue (reviewed in [38]). In the *mdx* model, first onsets of the disease occur around 2–3 weeks after birth but then, the disease slows down when mice are around 3-month-old. The amount of fibrotic replacement is also significantly lower and regenerative capacity seems to be much more potent [36]. Furthermore, in comparison to DMD patients, the lifespan of the *mdx* mice is not as significantly shortened, probably due to the much slower disease progression as many of the more serious symptoms occur later than in DMD [39]. Importantly, the first signs of cardiomyopathy (measured by echocardiography) appear around 10 months of age, whereas histological examination revealed interstitial cardiac fibrosis in 17-month-old mice [40]. Nevertheless, *mdx* mice still serve as the most frequently used animal model of DMD.

### Mouse model—*mdx*/utrn^−/−^ mice

Quite frequently used model relies on additional utrophin deficiency. Utrophin is a large, cytoskeletal protein which is very similar to dystrophin. In healthy muscle fibers, utrophin in strongly expressed only during the developmental stage and is further being replaced by the dystrophin [41, 42]. In adults, its appearance is limited to the vascular smooth muscles, nerves, endothelium, and neuromuscular junctions [41, 42]. Interestingly, utrophin was shown to be upregulated and constantly accumulated in regenerating muscle fibers of *mdx* mice [43]. Its occurrence might suggest some compensatory mechanism for the lack of dystrophin protein, especially because utrophin expression is normally down-regulated after the birth (reviewed in [44]).

Indeed, *mdx* mice additionally lacking utrophin have a more severe disease phenotype with progressive weight loss, muscle weakening and ultimately, they die prematurely at 20 weeks of age due to respiratory failure [45, 46]. Of note, whereas *mdx* single knockout mice do not exhibit signs of cardiomyopathy until 10 months of age [40], in *mdx/utrn*^−*/*−^ mice first symptoms of heart dysfunction, such as cardiomyocyte necrosis, appear at 8–10 weeks of age [46] and by 15 weeks of age decreased left ventricular fractional shortening and ejection fraction, as well as cardiac fibrosis, was observed [47].

### Mouse model—*mdx*/mTR^−/−^

Another interesting and very useful murine model is connected to the differences in the length of telomeres. As shown by many studies, despite their shorter lifespan, mice have much longer telomeres when compared to humans [48]. To see what will be the result of telomerase shortening in *mdx* mice, the special strain has been created. *mdx*/mTR^−/−^ mice possess a deletion in the RNA component TERC (mTR) of telomerase and they show more severe dystrophic phenotype than *mdx* mice which in many aspects resembles what is happening in DMD patients [49]. Sacco et al. [49] observed impaired self-renewal capacity of SCs, accumulation of fibrotic tissue, increased CK levels, dilated cardiomyopathy and also shortened lifespan (around 12 months) of *mdx*/mTR^−/−^ mice in comparison to *mdx*/mTR^+/−^ and wild-type mice. Importantly, defects in muscle regenerative response due to progressive exhaustion, impaired proliferation and engraftment potential of SCs necessary to fuel myofiber repair is a major consequence of telomerase shortening (reviewed in: [35]).

However, *mdx*/mTR^−/−^ and similarly *mdx*/utrn^−/−^ mice are not as frequently used as *mdx* animals, mostly because of the additional mutation, which makes them a much more complex genetic model than *mdx* mice.

### Canine model—golden retriever muscular dystrophy (GRMD)

Golden retriever muscular dystrophy (GRMD) is one of the best characterized DMD models to date. The advantage of GRMD over *mdx* mice is that the phenotype of the disease is much more similar to the human DMD. Relatively early age of the disease onset (visible even in 10-day-old dogs) and also increased the speed of symptoms progression in such animals have been observed [50, 51]. Notably, while the GRMD model seems to be more clinically relevant than the *mdx* mice, its usage is limited by the high costs of the experiment and the number of dogs that would be needed.

Despite the great deal of light that was shed on the subject of DMD pathology, there is still a need for the refinement, as none of known so far models exhibit the full range of human DMD symptoms while being good genetic equivalents.

## The role of angiogenesis in DMD—general overview

**Fig. 2:**
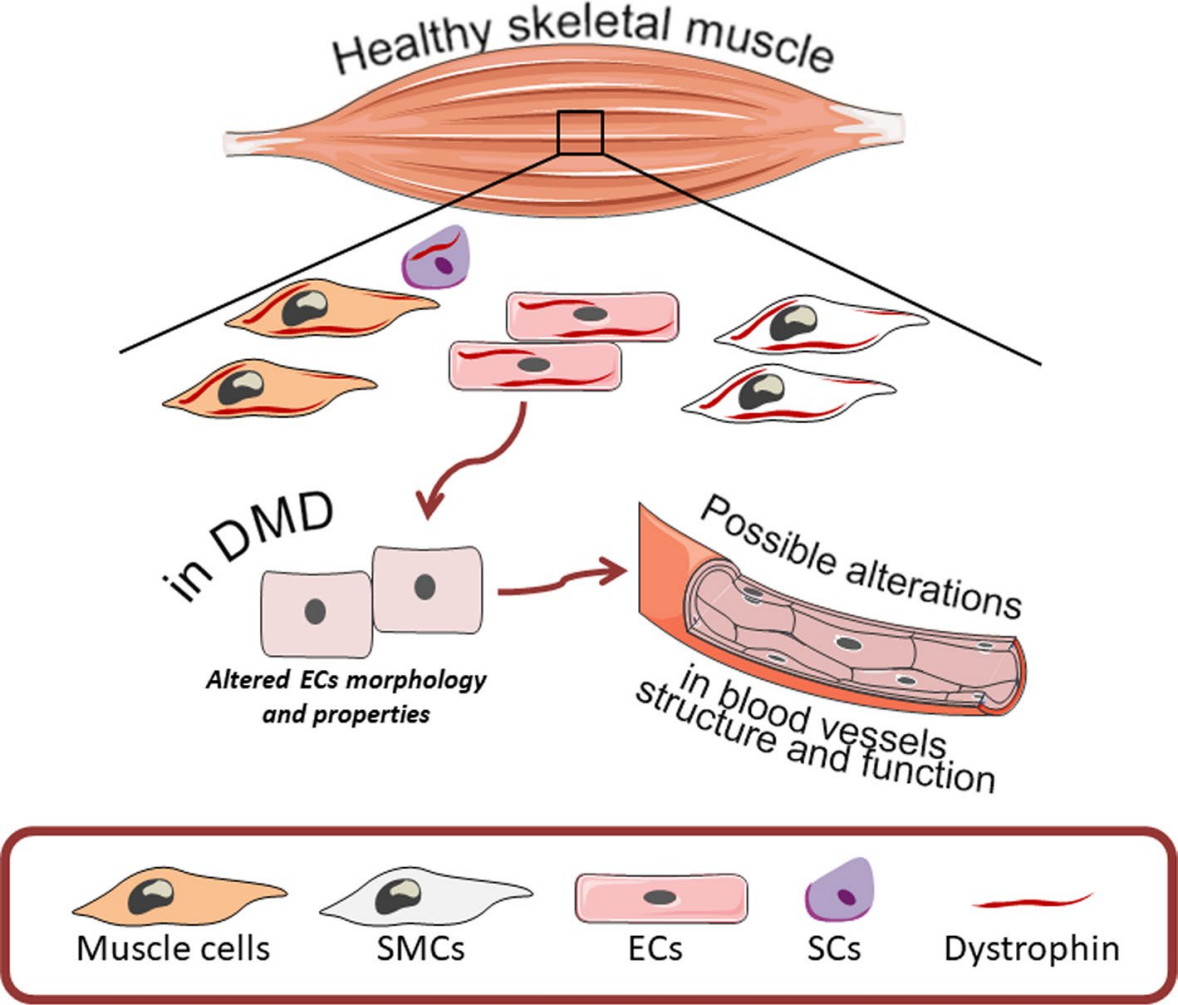
Possible alterations in angiogenesis process in DMD. Within the healthy skeletal muscle, dystrophin expression is not only restricted to muscle cells, but it is also present in other cell types including smooth muscle cells (SMCs), satellite cells (SCs) and endothelial cells (ECs). In DMD, the lack of dystrophin is associated with altered morphology and properties of ECs what potentially could lead to alterations in blood vessel structure and function

Several abnormalities have been suggested to affect DMD progression and additionally, the dysfunctional angiogenesis was also proposed to have an impact on DMD pathology. Angiogenesis, a process of new blood vessel formation from preexisting ones undeniably plays a pivotal role in muscles which comprise nearly 40% of the human body mass. Thus, the efficient delivery of oxygen as well as the transport of various metabolites is required for proper functioning of the muscles and thereby the whole body (reviewed in [52]). As mentioned earlier, dystrophin is expressed not only in muscle cells but is present also in SCs, vascular smooth muscle cells and endothelial cells [16, 53, 54] suggesting that in DMD patients the formation of blood vessels and the properties of endothelial cells might be impaired (Fig. 2). Indeed, already before the discovery of dystrophin by Kunkel et al. [2, 55], several studies aimed at the investigation of potential defects in vasculature which could be responsible for DMD pathology. As it was initially suspected, the presence of grouped necrosis in muscles of DMD patients could be explained by impaired capillary blood supply and ischemia [56, 57]. Although the vascular theory was extensively discussed, some studies undermined its validity, as no direct evidence for severe abnormalities in blood vessel morphology as well as in blood flow were observed [58–62]. Nonetheless, notable alterations, such as swollen and pale endothelial cells were revealed and their impaired function which could at least contribute to the pathogenesis of DMD was suggested [58, 62–64]. Moreover, Miike et al. [64] demonstrated aberrations in blood vessel structure in muscle biopsy specimens from DMD patients. Both the capillaries and endothelial cells area were much greater in DMD patients with almost completely occluded, narrow lumen in comparison to control subjects. Replication of the basement membrane around the vessels, as well as the presence of degenerating and regenerating capillaries, were also evident [64]. Such observations were further confirmed by the same group in Fukuyama-type congenital muscular dystrophy (FCMD) [65]. More recent studies focused on the analysis of vasculature in muscle tissues of *mdx* mice. Whole-mount imaging of tibialis anterior (TA) muscle, as well as immunostaining of arterioles in gracilis muscle, revealed marked decrease in the vasculature of *mdx* mice in comparison to wild-type animals [66, 67]. Additionally, altered biomechanical properties of carotid arteries in *mdx* and sarcoglycan-δ (sgcd^−/−^, a model of LGMD dystrophy) mice were also noticed [68]. All of the above strongly emphasize morphological aberrations of blood vessels both in mice models of DMD and in DMD patients, which potentially could have functional consequences.

### Evidence for angiogenesis alterations in animal models of DMD with respect to age

**Fig. 3:**
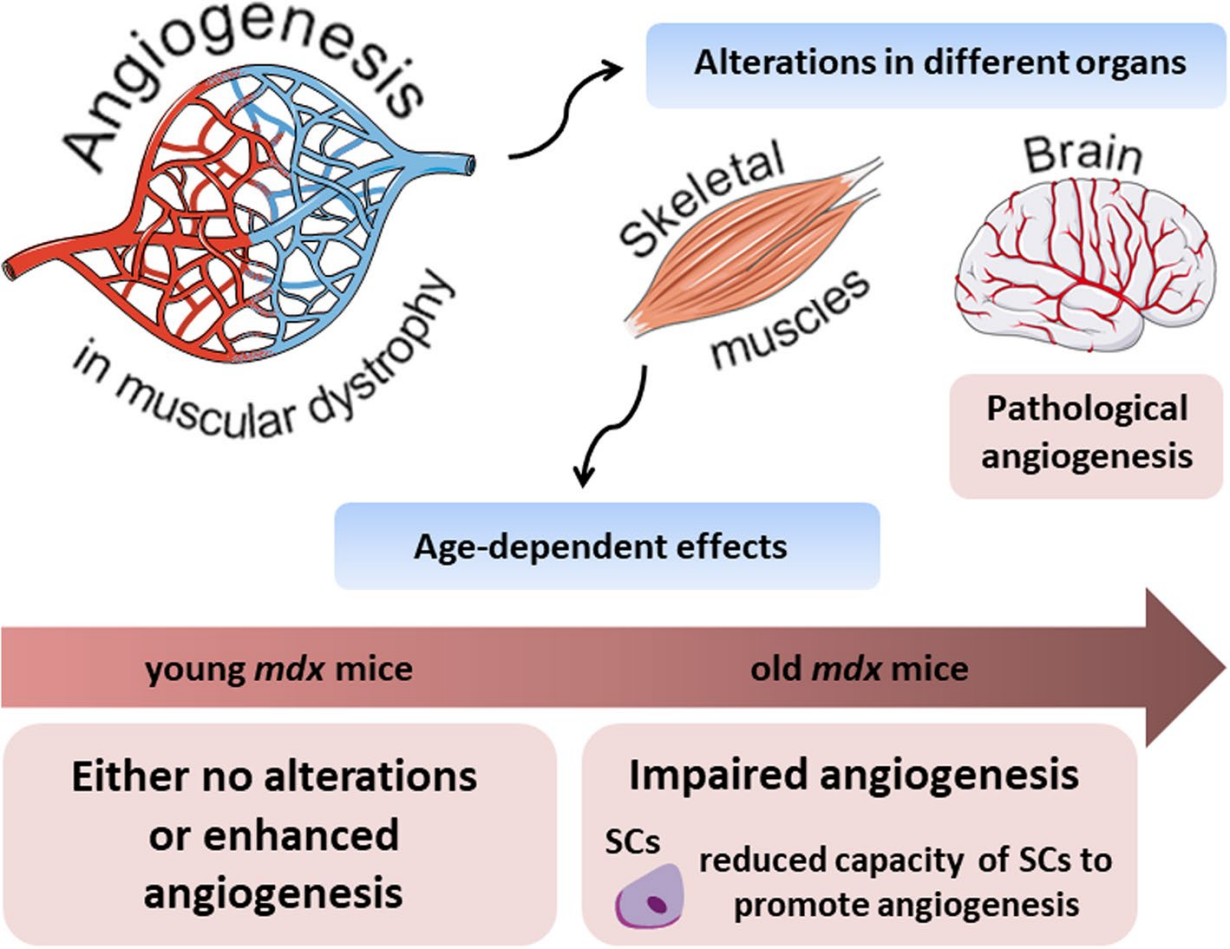
Age-related differences in angiogenesis process in dystrophic mice. Alterations in the angiogenesis process in muscular dystrophy are visible in different organs. In the brain of *mdx* mice, pathological angiogenesis with, among others, increased vascular permeability and abnormalities in cerebral perfusion were reported. On the other hand, in skeletal muscles, observed effects appear to be strongly age-dependent. In young, 2–3-month-old animals either no alterations or enhanced angiogenesis were documented; however, when older, 6–12-month-old mice were analysed, impairment in the angiogenesis process was evident. It was further suggested that such differences observed in old animals might be, at least partially, explained by the reduced capacity of satellite cells (SCs) to promote angiogenesis process

Interestingly, Straino et al. [69] showed the normal angiogenic response in a model of hindlimb ischemia in *mdx* mice. Moreover, even better arteriogenesis (increased arterioles but not capillaries length density) was observed in comparison to control animals even though no changes at the basal conditions were revealed. Angiogenic response to wound healing with concomitant increase in arterioles (but not capillaries) density was also observed in *mdx* mice in comparison to the wild-type counterparts [69]. Some contradictory effects have been described in the next studies. In 2013, Palladino et al. [70] reported the angiogenic impairment of the vascular endothelial cells isolated from *mdx* mice, including their migration, proliferation and tube formation in comparison to wild-type controls. The differences between the above-mentioned studies may be related to the age of animals which were used (Fig. 3). Straino et al. [69] examined young (2-month-old) *mdx* mice, which still have a quite potent regenerative capacity, whereas in the latter study older, around 6-month-old *mdx* mice, have been studied [70]. Importantly, the comprehensive analysis of age-dependent alterations of skeletal muscle microvasculature was performed most recently by Latroche et al. [71]. By utilizing specific mouse model: Flk-1^GFP/+^mice crossed with *mdx* mice (in which GFP is targeted in VEGFR-2 (Flk-1) gene locus) which enable visualization of all blood vessels and by applying innovative structural and non-invasive functional approaches such as 3D microvascular network organization and nuclear magnetic resonance (NMR), respectively, they compared muscle vasculature in young, 3-month-old versus old, 12-month-old dystrophic mice. In line with previous assumptions, although microvascular network organization in young dystrophic mice was not changed (except a slight decrease in terminal arteriole density), an increase both in muscle perfusion and mitochondrial oxidative phosphorylation was noticed. Oppositely, old mice displayed significant vasculature alteration with a concomitant reduction in muscle perfusion [71]. Additionally, age-related differences in muscle vasculature were also noticed in GRMD dogs. No differences in microvessel density, as well as total vascular area, were found in young, 1- to 3-month-old dystrophic dogs in comparison to control ones. Conversely, in older, 4- to 10-month-old dogs, microvessel depletion was observed; however, only in sartorius cranialis muscle [72].

It was further suggested that satellite cells, which are pivotal for regeneration process, can be, at least partially, responsible for impaired angiogenesis in aged mice, especially because they lie close to the capillaries and except their direct involvement in the myogenesis, they may also strongly influence angiogenic response, e.g., by secreting pro-angiogenic vascular endothelial growth factor (VEGF) [73]. Moreover, the close proximity of SCs to capillaries and thus the crosstalk between SCs and endothelial cells (mostly via Notch signaling) influences SCs self-renewal and quiescence [74]. Indeed, Rhoads et al. [75] have shown that SCs isolated from aged, 12-month-old *mdx* male mice exhibit reduced capacity to promote angiogenesis in vitro. The number, as well as the length of the sprouts in three-dimensional microvascular fragment model exposed to conditioned media (CM) from dystrophic mice, were significantly decreased as compared to the effect observed with CM from wild-type animals. Furthermore, the level of VEGF, as well as hypoxia-inducible factor-1α (HIF-1α) in dystrophic SCs, was significantly decreased in comparison to control cells, emphasizing their potential contribution to impaired angiogenesis process in dystrophic mice [75]. Restoration of VEGF expression was then proposed as a novel strategy to improve angiogenesis in DMD and the efforts made in this area will be discussed in more details below.

## The role of VEGF in DMD—pro-angiogenic and pro-myogenic effect of VEGF

It was suggested that one of the strategies to improve the dystrophic phenotype is to regulate the deficient angiogenesis in *mdx* mice. VEGF, a potent pro-angiogenic molecule, stimulating the migration, proliferation, and survival of endothelial cells is acting mostly through VEGFR-2 pathway [76]. Described as the endothelial-specific growth factor, VEGF was then found to be beneficial for many other cell types, including neurons and muscle cells [77, 78]. Importantly, it exerts direct myogenic effects and was demonstrated to have anti-apoptotic properties towards myogenic cells and was able to enhance muscle force restoration subsequent to traumatic injury [78, 79]. Moreover, muscle-specific deletion of VEGF was shown not only to dramatically decrease capillary density but also to reduce exercise endurance in mice [80, 81]. Concomitantly, it is known that VEGF mRNA level increases upon physical activity [82], which is greatly attenuated in DMD patients. Diminished level of VEGF associated with reduced angiogenesis and impaired growth of regenerated muscle fibers was also evident in *mdx*/MMP-2^−/−^ mice [83]. Hence, taking into account beneficial properties of VEGF related to both angiogenesis process as well as its involvement in myogenesis, it might be suspected that potential deficiency of VEGF in muscular dystrophy might contribute to the severe manifestation of the disease.

### The level of VEGF in DMD patients and in murine models of DMD

Evaluation of DMD patients’ and *mdx* mice blood revealed changes in VEGF level; however, the results coming from those studies are rather inconclusive. Saito et al. [84] found an elevated level of VEGF in the serum of patients suffering not only from DMD but also other, much milder dystrophies, such as BMD. However, in this study, male dystrophic patients were compared to much older control subjects of both sexes. In contrast to DMD patients, decreased level of VEGF was noticed in gastrocnemius (GM) [71] and soleus [85] muscles of *mdx* mice as well as in the diaphragm of *mdx*/utrn^−/−^ animals [86]. Moreover, transcriptome-based analysis of GM performed by our group revealed markedly diminished level of VEGF in *mdx* mice in comparison to wild-type animals (unpublished data). Interestingly, we and others showed that the level of microRNA-206 (miR-206), one of the key regulators of myogenesis (reviewed in [87]) is strongly upregulated in *mdx* mice of different ages in comparison to wild-type counterparts both in muscles and SCs [10, 88]. Except its direct and essential role in muscle development, miR-206 was also proposed to negatively regulate angiogenesis by repressing VEGF expression [89–92]. Notably, Bulaklak et al. [93] showed that AAV-mediated miR-206 inhibition not only improved overall motor function and attenuated dystrophic phenotype of *mdx* mice but was also associated with increased VEGF expression and concomitantly, induced angiogenesis activity in muscles. Altogether, impaired angiogenesis with a concomitantly diminished level of VEGF strongly suggests that VEGF restoration could potentially exert beneficial effects on DMD pathology.

### Different approaches to enhance muscle angiogenesis with regard to VEGF or VEGF-related pathways

**Fig. 4:**
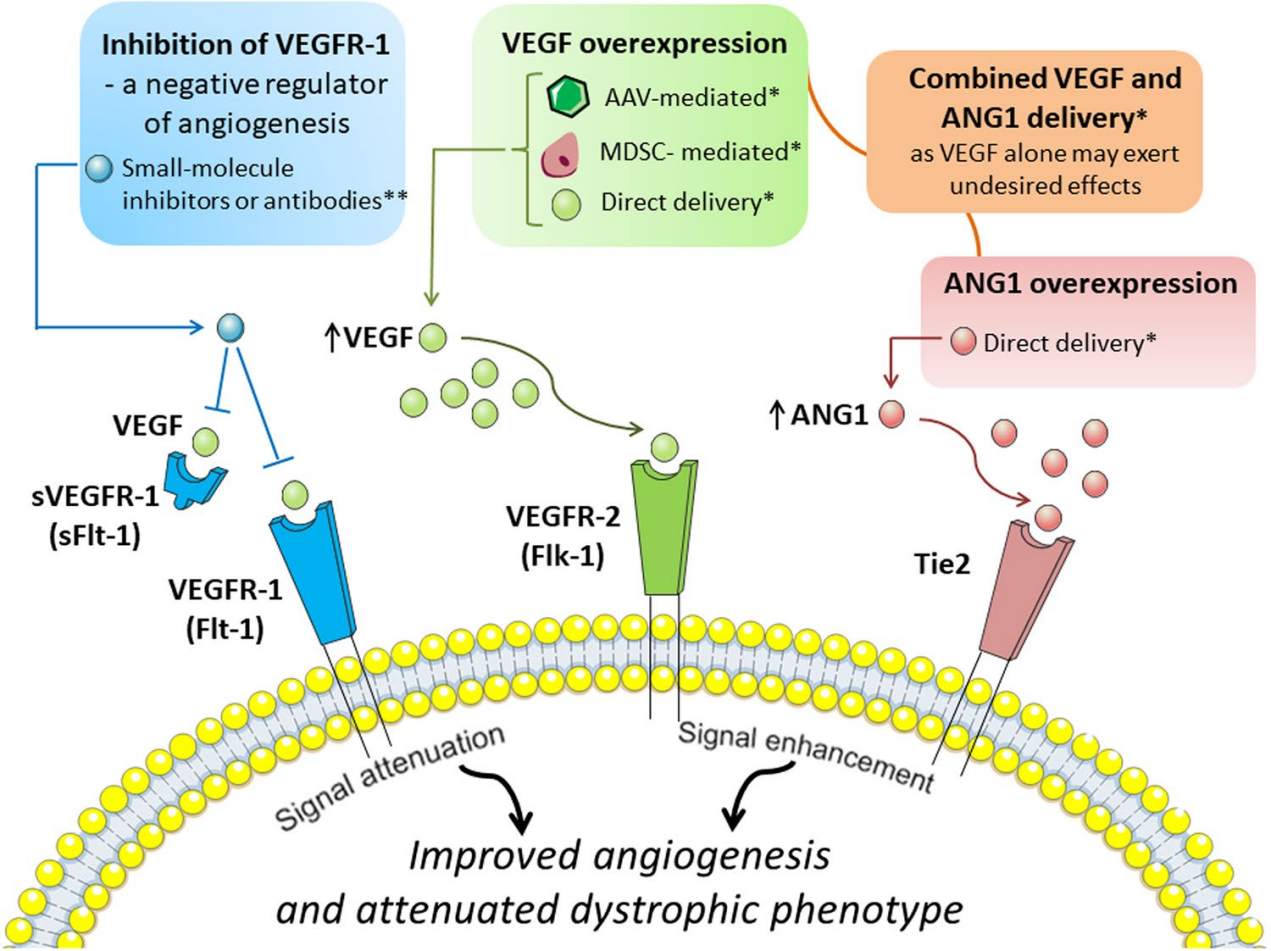
Angiogenic-dependent possibilities to improve dystrophic phenotype. Several angiogenic-dependent approaches to improve dystrophic phenotype were suggested. Among them, vascular endothelial growth factor-A (VEGF) overexpression obtained by several methods including adeno-associated adenoviral vectors (AAVs) harbouring VEGF, muscle-derived stem cells (MDSCs) engineered to overexpress VEGF or direct, intramuscular delivery of growth factor have been tested. Nonetheless, due to the fact that VEGF overexpression may exert undesired effects, angiopoietin 1 (ANG1) overexpression alone or combined delivery of VEGF and ANG1 was proposed to be more beneficial than administration of VEGF alone. Moreover, also inhibition of vascular growth factor receptor 1 (VEGFR-1) or its soluble form (sVEGFR-1), a negative regulators of angiogenesis, is considered to have a beneficial effect in DMD. *VEGFR-2* vascular endothelial growth factor receptor-2. *Tie2* tyrosine kinase with immunoglobulin-like and EGF-like domains 1. *Experimentally tested, **requires further validation as beneficial effects were reported only in mice haploinsufficient for VEGFR-1

Several tools can be applied to achieve VEGF overexpression (Fig. 4) (reviewed in: [94, 95], but adeno-associated viruses (AAV), especially serotype 9 (AAV9) are mostly used for muscle gene transfer and efficient transduction of skeletal and cardiac muscle after systemic administration [96]. In fact, Messina et al. [97] using AAV for intramuscular VEGF delivery to *mdx* mice were able to show increased regenerating fibers area with enhanced capillary density and reduced area of necrotic fibers. In another study, overexpression of VEGF (again using AAVs) in dystrophic mice led to decrease in serum CK level and what is more important, the measurement of grip strength showed the functional effect of VEGF therapy [98]. Although muscle regeneration was not changed after VEGF delivery, the area of inflammation and necrosis was significantly reduced when compared with saline-treated *mdx* mice. Despite the fact that these experiments clearly indicate the beneficial effect of VEGF overexpression, it has to be noted that control mice were injected with saline (not with control AAV vector) [98]. Importantly, concomitant, intravenous injection of VEGF and recombinant AAV6 vector harboring microdystrophin cassette significantly enhanced muscle-specific transduction efficiency in *mdx* mice, and thus, enabled widespread expression of functional microdystrophin at lower vector doses [99].

A different strategy to restore VEGF expression was demonstrated by Deasy et al. [100], who studied the effect of in vivo transplantation of muscle-derived stem cells (MDSCs) engineered to overexpress human VEGF. Local delivery of such cells into the GM of dystrophic and severe combined immunodeficient (*mdx*/SCID) mice resulted in marked increase in angiogenesis in comparison to mice injected with MDSCs control cells, as evidenced by immunostaining of microvasculature marker, platelet endothelial cell adhesion molecule 1 (CD31). However, it has to be emphasized that CD31 is also expressed by immune cells, which are abundant in dystrophic muscles. Furthermore, observed changes were accompanied by enhanced muscle regeneration and decreased fibrosis. Conversely, the opposite effects were reported when MDSCs expressing soluble forms-like tyrosine kinase 1 (sFlt-1, sVEGFR-1), a member of the VEGFR family and a negative regulator of angiogenesis with greater affinity to bind and sequester VEGF than VEGFR-2 [101], were transplanted [100]. Thus, one could suspect that inhibition of VEGFR-1/Flt-1 (or its soluble form) could result in improved angiogenesis, providing an alternative approach to VEGF overexpression. Indeed, Verma et al. [102] have reported that *mdx* mice developmentally haploinsufficient for Flt-1 (*mdx*:Flt-1^+/−^) exhibit overall improvement of dystrophic phenotype with increased muscle vasculature, decreased fibrosis and calcification in comparison to wild-type mice. Of importance, functional assays revealed increased muscle blood flow measured by the laser Doppler flow as well as ameliorated muscle contractile function in *mdx*:Flt-1^+/−^ mice as compared to control mice [102]. Although the results from this work are very promising, studies utilizing, e.g., small molecule inhibitors or antibodies specifically targeting Flt-1 should be used to fully address the question if inhibition of Flt-1 in postnatal life would be as beneficial as developmental lack of Flt-1 (summarized in Fig. 4).

### A critical view on the potential use of VEGF alone as a treatment option for DMD

It appears that utilization of VEGF as a potential treatment option for DMD patients has to be carefully considered, especially taking into account the dosage of VEGF being used, as its over-administration might exert severe adverse effects, ranging from edema or hemangioma formation [103, 104] to the development of endothelial cell-derived vascular tumors [105]. Additionally, due to the fact that systemic VEGF clearance proceeds rapidly [106], it might be hypothesized that beneficial effects of VEGF will be rather short-lived, and thus, frequent delivery of VEGF would be needed to sustain VEGF-induced changes both with regard to angiogenesis and myogenesis processes.

Furthermore, although several studies conducted in murine models of DMD pointed out advantageous effects of VEGF on the dystrophic phenotype, one cannot forget that *mdx* mice do not develop characteristic features of the disease to the same extent and as early as in humans [39]. Gutpell et al. [107] have investigated the effect of VEGF administration on fibrosis markers using heterozygous *mdx*/utrn^+/−^ mice lacking dystrophin and partially utrophin. Interestingly, the pro-fibrotic response of skeletal muscle fibroblasts isolated from 10-week-old *mdx*/utrn^+/−^ mice to VEGF administration, with increased *Acta2* expression encoding alpha-smooth muscle actin and enhanced stress-fiber formation, in comparison to untreated control fibroblasts was detected [108]. Moreover, also Gutpell et al. [86] in another study have shown that although expression of VEGF is significantly decreased in the diaphragm (but not in GM) of *mdx*/utrn^+/−^ mice, intramuscular, short-term delivery of low dose VEGF does not improve blood flow, as evidenced by applying dynamic contrast-enhanced computed tomography (DCE-CT). Hence, they undermined functional, pro-angiogenic effects of VEGF delivery and blunted an enthusiasm in the field of the potential use of VEGF alone as a treatment option for DMD. Instead, they revealed that the administration of another growth factor, angiopoietin 1 (ANG1) alone or in combination with VEGF significantly increased functional parameters of perfusion, such as blood volume. Additionally, VEGF treatment resulted in higher collagen deposition in comparison to ANG1-treated *mdx/*utrn^+/−^ animals and ANG1 induced vessel maturation in *mdx*/utrn^+/−^ hind limb muscle as compared to both sham-injected animals and VEGF-treated mice [86]. Thus, ANG1 might be another candidate for vascular-based therapy for DMD patients. Accordingly, combination delivery of VEGF with other factors, such as ANG1 or insulin-growth factor-1 (IGF-1) which was shown to promote muscle regeneration [109], rather than VEGF alone, should be considered (Fig. 4).

## The potential role of angiogenesis and VEGF in the heart muscle of murine models of DMD

Cardiac failure and dilated cardiomyopathy (DCM) are the common cause of death in patients suffering from DMD [110]. Although the involvement of angiogenesis process specifically in the heart has not been thoroughly investigated, it might be suspected that dystrophin deficiency, similarly like in case of skeletal muscles, will also affect angiogenesis in cardiac tissue. Indeed, Loufrani et al. [67] showed lower arteriolar density in the right ventricle of young, 12-week-old male *mdx* animals in comparison to control mice. However, some discrepancies were reported in another study done by Lai et al. [111], in which no differences in both numbers of arteries and the capillary area in the heart of old, female dystrophic mice were found. Nonetheless, except differences in age and sex of animals used, it has to be noted that in the latter study, *mdx* mice on C57Bl/10ScSn-*Dmd*^*m*dx^/J background were compared to the wild-type animals on C57Bl/10 background (and not the proper control—C57Bl/10ScSnJ), which utilization as controls might be questionable. Interestingly, downregulation of VEGF and decreased capillary density were already reported in the heart of patients with end-stage DCM [112]. Even more significant to this discussion is the fact that disturbed secretion of VEGF in the myocardium was observed in *mdx* mice exposed to hypobaric hypoxia in low-pressure chambers and such an effect was proposed to potentially promote the progression of the cardiomyopathy [113]. Of note, Chun et al. [114] revealed that aorta-derived mesoangioblasts (ADMs) differentiated into cardiomyocytes in vitro and injected into the heart of young *mdx*/utrn^−/−^ mice were able to prevent the onset of cardiomyopathy with a concomitant increase in the number of capillaries. Although this beneficial impact of ADMs transplantation on angiogenesis was claimed to be rather an indirect effect [115], it cannot be excluded that modulation of angiogenesis and VEGF expression could have a beneficial effect on DMD progression also with regard to the heart function.

## Pathological angiogenesis in the brain of murine models of DMD

In addition to the well-described pathological features of DMD directly related to the muscle weakness, DMD affects also a central nervous system (CNS) leading to the mental retardation in one-third of patients suffering from DMD [116]. Metabolic abnormalities in *mdx* mouse brain were reported in 1996 by the group of Tracey et al. [117]. Later, Nico et al. [118] have demonstrated for the first time that the microvessels area positive for VEGFR-2 as well as the area of neurons positive for VEGF in brain samples from 18 to 20-month-old *mdx* female mice were significantly elevated in comparison to control ones. Increased vascularisation of *mdx* mouse brain was further confirmed by the same group in another study, in which increased expression of VEGFR-2 and VEGF in younger, 5-month-old female *mdx* mice was correlated with the elevated level of HIF-1α and altered expression patterns of endothelial tight junction proteins zonula occludens-1 (ZO-1) and claudin-1 [119]. Tryptase, nerve growth factor (NGF), matrix metalloproteinases-2 and-9 (MMP-2 and -9) were also suggested to be involved in those alterations [120, 121]. Moreover, detailed analysis revealed that the majority of microvessels in dystrophic mice were coated with abnormal endothelial cells with open tight junctions [122]. Blood brain barrier (BBB) breakdown and increased vascular permeability in the brain of *mdx* mice were observed not only in adult mice but were already visible in dystrophic embryos as well as newborn mice [123]. Additionally, to the results obtained by the group of Nico et al. [118], an elegant study performed recently by Goodnough et al. [124] demonstrated, for the first time, functional abnormalities of cerebral diffusion and perfusion in male *mdx* mice at a different age. A comprehensive combination of in vitro, in vivo and ex vivo analysis of *mdx* brain, revealed disruption of BBB integrity and decreased cerebral diffusivity both in 2- and 10-month-old *mdx* mice in comparison to wild-type animals. Furthermore, 10-month-old *mdx* mice exhibited enhanced cerebral arteriogenesis and, most strikingly, diminished cerebral perfusion [124]. Such alterations might be related to the cerebral edema formation and impaired functioning of water channels in the brain of *mdx* mice [125]. Altogether, pathological angiogenesis in the brain of dystrophic mice can be associated with the development and progression of neurological dysfunction found in DMD patients.

## Sex influences dystrophic phenotype and angiogenesis process

DMD affects mainly boys, but symptomatic female carriers, with the variable manifestation of the disease symptoms, are also described [126–128]. Preclinical studies on animal models of DMD investigate mostly male mice, but some were performed without respect to sex [129, 130] or were done solely on female mice [111, 118, 119]. Nonetheless, it might be envisaged that the dystrophic phenotype differs with respect to the sex; thereby the studies evaluating DMD progression in female *mdx* mice might not represent a reliable model of DMD. Indeed, a substantial number of studies suggest that the sex of dystrophic mice have a huge influence on the disease outcome. Shortly, it appears that young female *mdx* mice exhibit attenuated dystrophic phenotype in comparison to male *mdx* mice; however, this effect is strongly reversed with age [131–133].

Moreover, sex-related alterations are not only restricted to skeletal muscles but are also manifesting in cardiac function. Female *mdx* mice at 22 months of age exhibit much more prominent cardiac dysfunction in comparison to male mice, with, among others, significantly worse hemodynamic function and reduced ejection fraction [134]. Hence, the utilization of old female *mdx* mice as a model to study DMD-related cardiomyopathy was suggested [111, 135–137].

Finally, unexpected, sex-related alterations in angiogenesis process have been reported recently by Guéniot et al. [138] who studied the vascular network in 12-month-old female *mdx* mice. Complex methodology, including histological assessment as well as functional approaches, such as non-invasive NMR-enabled broad and reliable investigation of the angiogenesis process in female dystrophic mice. Strikingly, the authors showed enhanced reperfusion after ischemic stress followed by increased vascular density and elevated mitochondrial oxidative rephosphorylation capacity in female *mdx* animals in comparison to wild-type counterparts. Obtained results are completely opposite to the effects observed by the same group in age-matched male *mdx* mice [71]. The differences observed in males and females might be at least partially regulated by sex hormones as estragon and estradiol have been shown to enhance angiogenesis, through increased endothelial cells proliferation and migration. This effect might be mediated by the upregulation of endothelial nitric oxide synthase (eNOS) activity, increased VEGF level and adhesion molecules expression (reviewed in: [139]).

All of the above strongly emphasize sex-related differences in key aspects of DMD pathology, pointing out the possible contribution of female-specific factors, such as sex hormones, to those alterations [140, 141]. Altogether, sex has to be carefully considered when designing experimental settings utilizing dystrophic mice. It has to be pointed out that the effects observed in female *mdx* mice, also with respect to angiogenesis process, might not be biologically relevant when considering the disease which affects boys.

## Other possible modulators of angiogenic status in DMD

As DMD is still an incurable disease, many studies concentrate on finding new molecular players having the capacity to modulate disease progression. Some selected factors are presented below.

### Nitric oxide and DMD

**Fig. 5:**
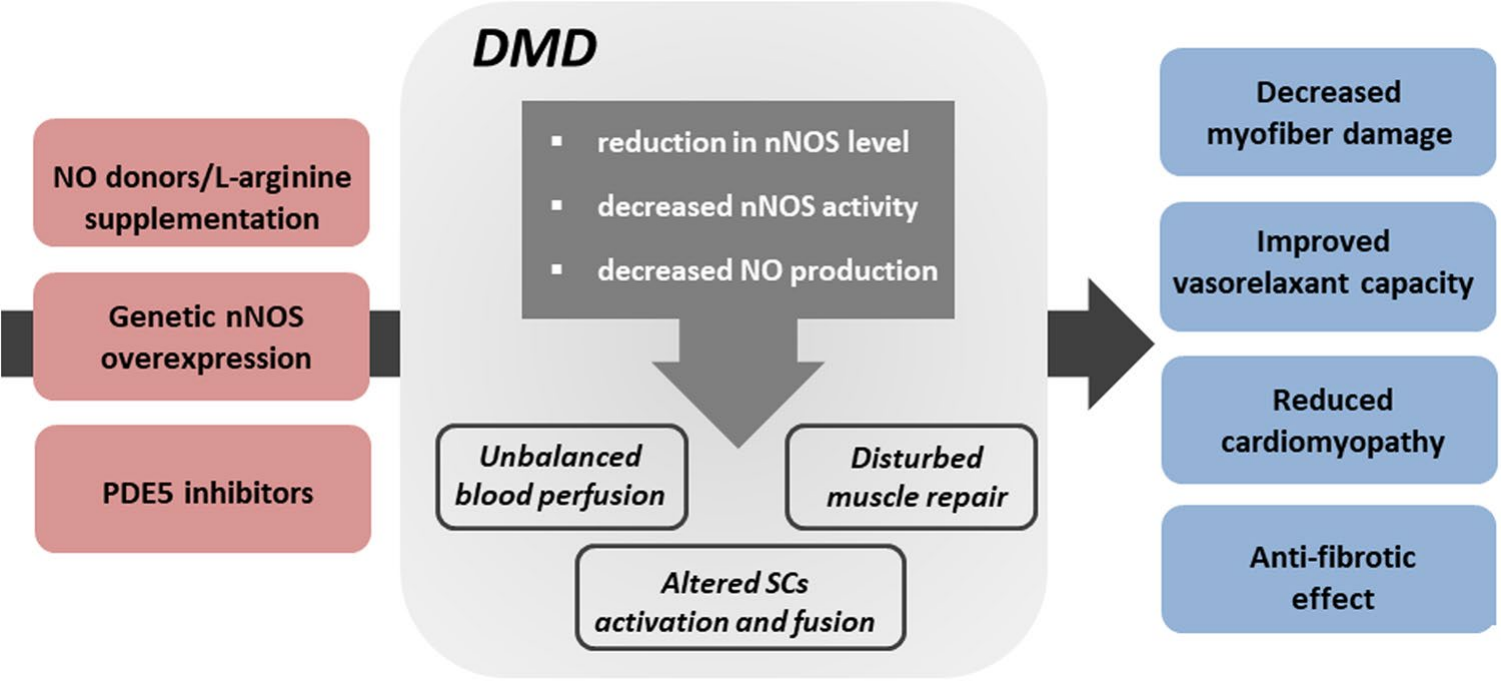
Impairment of NO pathway in the progression of DMD and therapeutic strategies to increase NOS activity and NO production. Neuronal-type nitric oxide synthase (nNOS) is a component of dystrophin complex and synthesizes freely diffusible nitric oxide (NO), a key signaling molecule. In dystrophic muscle, the dissociation of nNOS from the sarcolemma and subsequent reduction in nNOS level, nNOS activity, and NO production may contribute to the progression of dystrophic phenotype affecting not only blood perfusion but also muscle repair processes and satellite cells (SCs) properties. Thus, modulation of NO signaling pathway either via l-arginine and NO donor supplementation, genetic nNOS overexpression or 5-phosphodiesterase (PDE5) inhibitors was speculated to serve as a possible therapy for DMD. Indeed, a substantial number of studies revealed beneficial effects of such approaches on dystrophic phenotype, with decreased myofiber damage, improved vasorelaxant capacity, reduction in cardiomyopathy and anti-fibrotic effects

As mentioned earlier, dystrophin serves as a cytoskeleton stabilization protein but it also may play a signaling role, through the localization of signaling proteins. Neuronal-type nitric oxide synthase (nNOS) in skeletal muscle was demonstrated already many years ago to be a component of dystrophin complex [142]. nNOS synthesizes freely diffusible nitric oxide (NO), a key signaling molecule regulating blood flow, contraction, satellite cell activation, Ca^2+^ handling, mitochondrial biogenesis and gene expression [143]. In normal, healthy muscles, NO, among other activities, may modulate contractile force [144], regulates exercise-induced glucose uptake [145] and is implicated in myofiber differentiation [146]. In dystrophic muscle, the dissociation of nNOS from the sarcolemma and subsequent reduction in nNOS mRNA and protein level [147], nNOS activity [148] and NO production [149] may contribute to the progression of dystrophic phenotype (Fig. 5).

Furthermore, dystrophin regulates NO production not only by providing the scaffold for nNOS but also by the modulation of flow (shear stress)-mediated endothelium-dependent dilation (FMD) in arteries. Loufrani et al. [67] reported that the lack of dystrophin induces alterations in flow-dependent mechanotransduction with significantly decreased vascular density in gracilis and cardiac muscles in *mdx* mice in comparison to control animals. Of importance, these changes were attributed to significantly lowered NO-dependent FMD and eNOS expression [67]. The role of eNOS (as well as nNOS) in vascular response in contracting fast-twitch skeletal muscle is well known [150]; however, the status of eNOS in DMD has not been well investigated and the results vary between different studies. In *mdx* mice, eNOS expression in skeletal muscles does not seem to be changed on mRNA level [83], but on protein level either no changes [151, 152] or even elevation of eNOS was reported [153]. On the other hand, in a canine model of DMD, the vascular endothelial dysfunction with diminished eNOS protein level was reported [154]. Although the discrepancies between various studies are apparent, the increase in eNOS level achieved by different approaches was reported to exert beneficial effects in DMD [88, 154, 155].

NO is a potent modulator of vascular tone and muscle-derived NO plays a role in the regulation of blood flow. This action relies on the modulation of vasoconstrictor response to activation of α-adrenergic receptors. However, this mechanism is defective during contraction of nNOS knockout mice and nNOS-deficient *mdx* mice [151]. What is more important; similar results were observed in DMD patients. Sander et al. [156] have found that in DMD dystrophic boys, expression of nNOS in muscles is greatly reduced in comparison to children with polymyositis (PM) or LGMD. As the consequence vasoconstrictor responses were not attenuated in exercising forearm in children with DMD but were greatly decreased in those with PM or LGMD [156]. These data suggest that due to low production of NO by contracting human DMD skeletal muscles, a protective mechanism that blunts the α-adrenergic vasoconstrictor response to reflex sympathetic activation is defective and it leads to functional muscle ischemia when the dystrophic muscles are exercised [156].

Not only blood perfusion but also SCs activation and fusion leading to better muscle repair might be regulated by NO [157]. In our hands, in *mdx* SCs with impaired differentiation potential, the downregulation of NO-related cGMP-PKG (cGMP-dependent protein kinase) signaling pathway was observed. Moreover, supplementation with NO donor normalized the in vitro differentiation rate of SCs from *mdx* mice [10]. Moreover, NO may act as an epigenetic regulator in DMD—loss of nNOS can impair muscle development and regeneration by disrupting the normal regulation of chromatin structure [158]. It was also found that NO, through modification of histone deacetylases activity (e.g. S-nitrosylation of HDAC2) may affect the expression of some microRNAs, important for muscle regeneration (reviewed in [159]). Additionally, NO released from muscles may activate MMPs, especially MMP-2 [160], triggering the release of some trophic agents, such as hepatocyte growth factor (HGF), known to activate satellite cells through binding to c-met receptor [161]. Collectively, these mechanisms are not fully functional in dystrophic muscles and might be responsible for improper muscle growth and regeneration (Fig. 5).

### The possible treatment of DMD—modulation of NO and NO-dependent pathways

As nNOS level is greatly reduced in DMD patients as well as skeletal-derived NO production is blunted, and taking into consideration multiple mechanisms controlled by NO, it was speculated that modulation of NO signaling pathway might serve as a possible therapy for dystrophy treatment (Fig. 5). Several studies have revealed a beneficial effect of nNOS overexpression in dystrophic mice as the reduction in inflammatory reaction with decreased macrophage and neutrophil infiltration [162] as well as anti-fibrotic effect in dystrophic mice was observed [163]. Reduced macrophage content, reduced fibrosis, and increased survival rate after nNOS overexpression have been demonstrated also in *mdx*/utrn^−/−^ mice [164].

In addition, l-arginine, the substrate for nNOS, has been proposed as a pharmacological treatment to attenuate the dystrophic pattern of DMD. Hnia et al. [165] reported that 5-week-old male *mdx* mice treated with 200 mg/kg l-arginine for 2 weeks displayed lower inflammation (decreased NF-κB activation and lower IL-1β, IL-6 and TNFα level) and stabilization of utrophin/β-dystroglycan interactions as well as induction of the recruitment of nNOS in the sarcolemma. In contrast, long-term supplementation (17 months) with arginine failed to protect *mdx* mice, as increased fibrosis in muscles and hearts was observed [166]. In this study, *mdx* and C57BL/6 (used as controls) mice received drinking water supplemented with 5 mg/ml of l- or d-arginine from 3 weeks to 18 months of age. Intense fibrosis was observed after treatment with l- but not d-arginine, and only in *mdx*, not in control mice [166]. Additionally, NO donors including diethylenetriamine (NONOate, DETA-NO) [167] or guaifenesin dinitrate (GDN) [168] have been shown to reduce disease progression and increasing muscle function in dystrophic mice by regulating SCs activation.

Several studies described the use of arginine butyrate, the compound combining two pharmacological activities which contribute to DMD progression—NO-pathway activation by arginine and HDAC inhibition by butyrate. Intraperitoneal administration of arginine butyrate to *mdx* mice resulted in the improvement of the dystrophic phenotype in skeletal muscles and diaphragm both in newborn and adult mice [169]. The same group has also evaluated the oral administration of arginine butyrate and was able to demonstrate the improvement of cardiac function, decreased kyphosis and neuronal changes in treated *mdx* mice [170]. It should be also noted that Guerron et al. [171] evaluated long-term therapy with arginine butyrate (treatment for 6 months, beginning at 3 months of age for 5 days each week) and some tendency to improve grip strength and decreased fibrosis in the GM was observed, however, no significant changes in other parameters such as muscle histology, behavioral measurements, heart function or serum CK levels were detected. However, additional experiments are needed to fully understand the mechanism of this drug (proposed for the treatment of sickle-cell disease and β-thalassemia [172, 173]) in DMD patients.

Improved vasorelaxation capacity might be also suggested as the therapy for DMD. NO acts mainly by the activation of soluble guanylyl cyclase (cGS) to increase the level of cyclic guanosine monophosphate (cGMP) and then protein kinase G is activated to induce vasodilation. Therefore, 5-phosphodiesterase (PDE5) inhibitors, such as tadalafil and sildenafil, which inhibit cGMP degradation, might have a beneficial role in DMD treatment. Of note, the activity of PDE5 in *mdx* skeletal muscles is much higher than in control individuals [174, 175] but this regulation might be also age-dependent [176]. Young *mdx* mice had higher cAMP and cGMP PDE activity in comparison to age-matched controls; however, the older dystrophic animals (15 weeks of age) had similar cGMP PDE activity and lower cAMP PDE activity than controls [176]. Nevertheless, many studies have shown the usefulness of PDE5 inhibitors in mouse models of DMD. Treatment with sildenafil ameliorated the age-related cardiomyopathy [177] and reduced diaphragm weakness and fibrosis [178] in the *mdx* mice. Of note, the use of a PDE4 inhibitor, piclamilast as well as a combination of PDE4 and PDE5 inhibitors also exerted beneficial, anti-fibrotic effects in *mdx* mice [179]. Finally, in DMD boys, PDE5 inhibition alleviated exercise-induced skeletal muscle ischemia and was suggested to be a putative new therapeutic strategy for DMD [180].

Although many studies show positive effects of NO supplementation/modulation of NO signaling pathway in dystrophy pathology, there are also studies indicating that such treatment should be used with caution. Supplementation of dystrophic animals with sodium nitrate (85 mg/l) in drinking water had detrimental effects on skeletal muscle, potentially via ONOO^−^—dependent mechanism [181].

### The role of heme oxygenase-1 in angiogenesis and the possible role in DMD

One of the possible therapeutic agents playing a protective role in DMD might be heme oxygenase-1 (HO-1, encoded by *Hmox1* gene). This microsomal enzyme is responsible for the first, rate-limiting step in heme degradation pathway and it leads to the formation of carbon monoxide (CO), ferrous iron (inducing the synthesis of protective ferritin) and biliverdin (subsequently converted into anti-oxidant bilirubin by biliverdin reductase). HO-1 is a master cellular cytoprotectant, not only because it deactivates deleterious properties of pro-oxidant heme, but mostly due to many non-canonical functions, including anti-apoptotic, anti-inflammatory and anti-oxidant properties (for review see: [182–184]). We have shown its crucial role as blood vessel formation regulator and we demonstrated that angiogenesis induced by VEGF [185, 186] or SDF-1 [187] depends on HO-1. We underlined its importance for the proper wound healing process [188], in tumorigenesis [183, 189–192] and in the functions of bone marrow-derived pro-angiogenic cells [193, 194].

Recently, we focused on the role of HO-1 in the muscles (progenitor) cells biology, especially in the aspects of their regeneration potential after both acute injury and persistent muscular degeneration. We have shown that in non-dystrophic mice, short-term expression of HO-1 promotes myoblasts proliferation and muscle regeneration [195] while oppositely, its long-term expression inhibits differentiation, affecting significantly muscle-specific microRNAs (myomirs) [196]. We have revealed a role for HO-1 in the progression of *rhabdomyosarcoma* (RMS), a soft tissue cancer characterized by impaired myogenic differentiation [197]. Interestingly, we have also reported the opposite regulation between HO-1 and miR-206 in RMS cell lines and clinical primary tumors of the more aggressive alveolar type [197]. In a model of cardiotoxin-induced muscle damage, we have demonstrated increased muscle injury, disturbed proportion of M1/M2 macrophages, enhanced formation of arterioles and hypertrophy with the augmented rate of regeneration observed in injured HO-1 knockout skeletal muscle [198]. We have also found that HO-1-deficient satellite cells are prone to activation and have a higher proliferation rate after injury, suggesting that HO-1 is an important regulator of muscle repair mechanisms preventing its uncontrolled acceleration and hence exhaustion [198].

Our recent studies concentrated on the role of HO-1 in DMD progression [10]. Using *mdx* mice additionally lacking HO-1 (*mdx*/HO-1^−/−^), as well as by applying pharmacological inhibitor of HO-1 activity in *mdx* mice, we observed increased damage and inflammation in comparison to control mice. What is important, lack of HO-1 affected also running capacity of dystrophic mice—*mdx*/HO-1^−/−^mice ran shorter distance than *mdx*. We also observed disturbed and enhanced differentiation of satellite cells isolated from *mdx*/HO-1^−/−^mice. Finally, we have found that differentiation of *mdx* satellite cells might be normalized by supplementation of the cells with CO, a product of HO-1 activity as well as nitric oxide (NO) [10], suggested to have beneficial effects in DMD as discussed earlier. These data implicate HO-1 as a possible therapeutic target to reduce the progression of DMD.

### Statins—pleiotropic drugs regulating processes involved in DMD progression

Discovered 40 years ago [199], 3-hydroxy-3-methylglutaryl coenzyme A (HMG-CoA) reductase inhibitors, more commonly known as statins, are lipid-lowering drugs for the treatment of hypercholesterolemia and reduction of atherosclerosis. Noteworthy, statins comprise many subtypes based on structural differences and their pharmacokinetic properties, including their active or lactone form, lipophilic/hydrophilic rate, and their absorption and metabolism, what may affect some (slight) different effects exerted by various members of the family (reviewed in [200]).

Statin interaction with HMG-CoA reductase obtained already at nanomolar concentrations, leads to the inhibition of the downstream cholesterol biosynthesis and numerous isoprenoid metabolites such as geranylgeranyl pyrophosphate (GGPP) and farnesyl pyrophosphate (FPP). GGPP and FPP are key intermediates for post-translational modification of several cell signaling proteins, including the small GTPase family members Ras, Rac, and Rho. The isoprenylation is fundamental for the proper functioning of these proteins as regulators of cell shape, motility, differentiation, and proliferation (reviewed in [200]), therefore inhibition of Ras, Rho, and Rac is suggested to be the major targets responsible for pleiotropic effects exerted by statins [201].

The anti-fibrotic role of statins is one of the most commonly observed in different pathological conditions, including cardiac [202] and hepatic fibrosis [203]. Another, not connected to the lipid-lowering role is attributed to statins anti-oxidant properties. In 2012, Pignatelli et al. [204] showed that atorvastatin treatment in patients with polygenic hypercholesterolemia exerts anti-oxidant properties by inhibition of NADPH oxidase 2 (Nox2), which is one of the reactive oxygen species (ROS) generating enzyme. Moreover, in a dose-dependent manner, through increased endothelial NOS (eNOS) mRNA stability and protein activity, statins are able to increase NO bioavailability [205, 206]. Augmented NO level, by its anti-oxidant properties might itself decrease oxidative stress [207]; however, the more important role might be associated with regulation of angiogenesis. We have demonstrated that atorvastatin at a pharmacologically relevant concentration (100 nM) enhanced the expression of eNOS in human microvascular endothelial cells (HMEC-1). Moreover, atorvastatin prevented the hypoxia-induced decrease in eNOS expression [208]. The regulation of several angiogenic factors was observed by us after statin stimulation in HUVEC cells [209]. However, angiogenic activity of statins seems to be cell type and dose-dependent as shown in several (including ours) papers. It was demonstrated that at low, therapeutic (nanomolar) concentrations statins are pro-angiogenic, whereas at high dosage (low micromolar) we can observe the inhibition of angiogenic activity [209–211].

As mentioned earlier, statins inhibit Ras prenylation and this effect may result in downregulation of activity of NF-κB, which is important for many different inflammatory pathways [212] and is strongly activated in DMD [213]. In this aspect, statins were shown to downregulate the level of inflammatory mediators, such as monocyte chemoattractant protein-1 (MCP-1) and tumor necrosis factor alpha (TNF-α) [214].

### Statins in DMD—potential therapeutic agents?

For simplicity, DMD can be characterized by three main symptoms: chronic inflammation, oxidative stress, and fibrosis. Based on the statin pleiotropic effects that were described above, it seems that they could be considered as a good candidate to be used as therapeutic agents.

Interestingly, Whitehead et al. [215] for the first time described the protective effect of statins in *mdx* mice. The authors showed that simvastatin treatment at moderate daily dose improved muscle health, reduced inflammation, fibrosis, and oxidative stress. Treatment had no effect on the CK level, which increase is one of the markers associated with statin-induced myopathy. Moreover, the author decided to take a look at the most affected by the disease muscle, diaphragm, showing improvement in muscle strength and physiological performance. What is even more interesting, increase in muscle force of different muscles, reached about 40%, what is similar to the effect obtained by the most promising so far therapies—with mini-dystrophin construct and exon skipping [216, 217].

Another possible mechanism of statin action was connected to the regulation of autophagy, which is strongly impaired during DMD progression [14]. Simvastatin treatment was found to enhance this process in *mdx* mice and it was already shown that increased autophagy leads to a decrease in inflammation and fibrosis [14]. Impaired autophagy might be caused by CYBB/Nox2-mediated ROS production [218]. As mentioned before, statins decrease the level of Nox2, what strongly suggests that this pathway might contribute to intensified autophagy process [204].

DMD is a very progressive disease, starting at early age and the diagnosis is in many cases overdue and therapy is initiated when the irreversible symptoms already occurred [219]. Whitehead et al. [215] administrated simvastatin to old *mdx* mice and for the first time showed reversion of preexisting fibrosis in diaphragm muscle, suggesting that this drug is able to work on the already established disease. Based on all presented data one can claim that the therapeutic application of statins should be taken into consideration; however, no additional studies in this area are available so far. On the other hand, some work underlines the unfavorable effects of statins.

### Statins—their devastating (?) role in muscles

One of the suggested side effects of statin treatment is related to the induction of skeletal muscle myopathy and rhabdomyolysis, characterized by muscle breakdown and the release of intracellular content including CK and many electrolytes, being well-documented and most frequently described [220, 221]. Although several mechanisms responsible for adverse statin-induced effects were published, this issue is a matter of ongoing debate.

Several in vitro studies showed toxic effects of statins. However, very often the concentrations used were far beyond the physiological range, e.g., 24 h stimulation with 10 μM or 50 μM simvastatin, atorvastatin or rosuvastatin was performed in C2C12 myoblast cell line [222] or even 100 μM cerivastatin, fluvastatin and atorvastatin was used to induce cell death and mitochondrial toxicity in L6 rat skeletal muscle cell line [223]. Importantly, however, such high concentrations of statins required to induce deleterious effects in vitro are typically greater than 1 μM. Such concentrations are considerably (100–1000 times) higher than those found in vivo in mice and humans [224]. Therefore, a detailed analysis of the dose of statins used in various experiments is needed.

There are also discrepant data about the incidence of different kinds of myopathy in humans after statin therapy. Previous studies indicated a high risk of such adverse effects, e.g. showing that > 10% of statin users in the general population can be affected [225]. However, a recent systematic review of clinical trials found adverse muscle symptoms only in < 1% compared with placebo controls [225]. Statin-related muscle symptoms also appear to be exacerbated by several factors, including exercise, older age and female sex [226, 227]. Moreover, when statin-induced rhabdomyolysis cases were analyzed, it appeared, that in many patients, statins were used together with other drugs that could interfere with statins metabolism and in some cases potentiated the risk of negative side effects, such as rhabdomyolysis [220, 221].

**Fig. 6:**
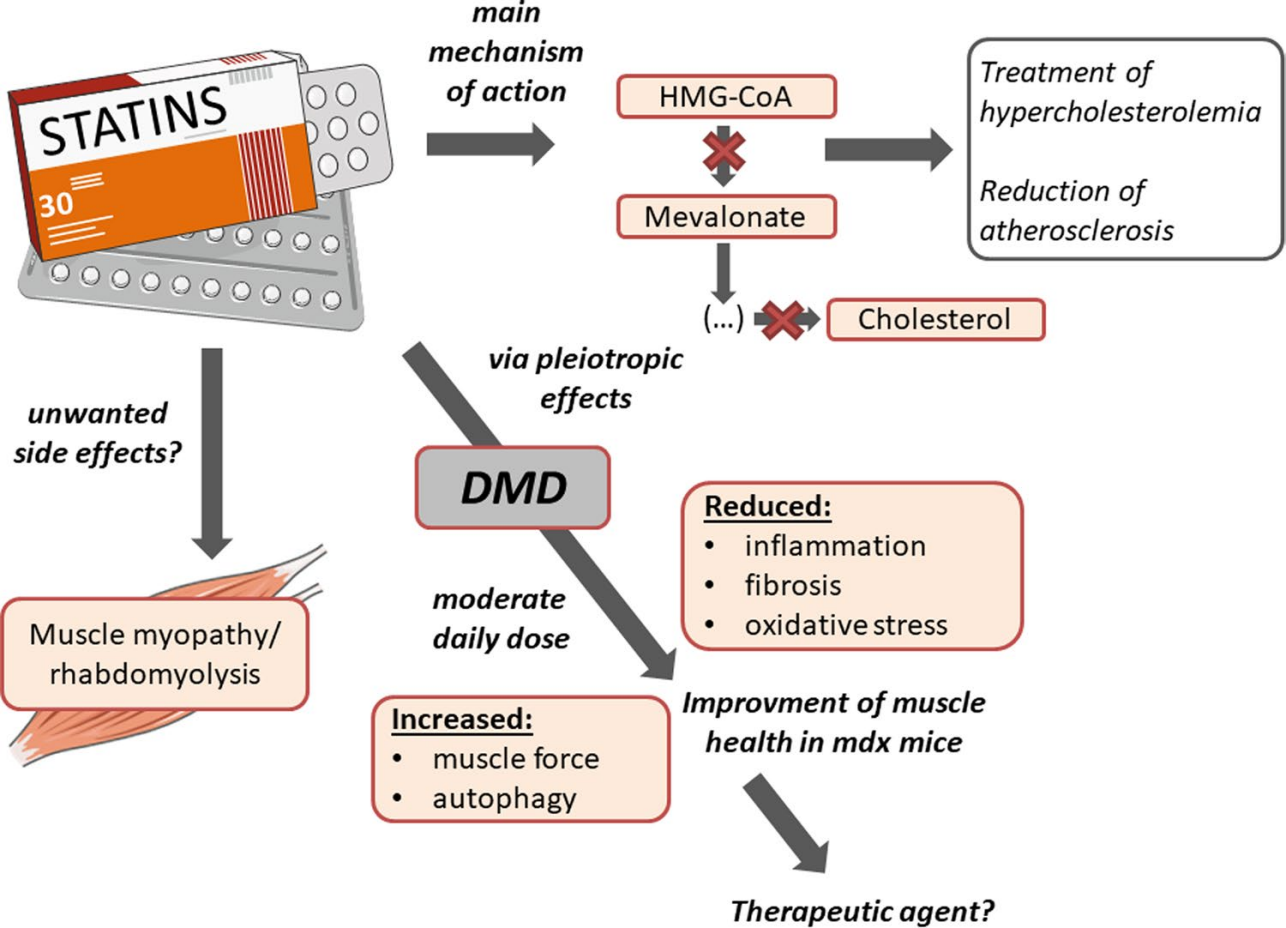
Statins as a potential therapeutic agent for DMD. Statins being 3-hydroxy-3-methylglutaryl coenzyme A (HMG-CoA) reductase inhibitors for many years were used as lipid-lowering drugs for the treatment of hypercholesterolemia and reduction of atherosclerosis. Nevertheless, via their pleiotropic actions, they may exert many other effects such as reduction of inflammation, fibrosis and oxidative stress that were shown to improve muscle health in *mdx* mice, suggesting the therapeutic application of statins. However, due to potential but recently strongly questioned the risk of unwanted muscle-related side effects including rhabdomyolysis, statins were ignored when looking for possible DMD treatment

Nevertheless, a recent meta-analysis by Iwere and Hewitt clearly showed that even in aged patients (65 + years), the risk of statin-induced myopathy was comparable to placebo patients [228]. These data implicate that the fear of statin-caused myopathy might be overestimated. Notably, the risk factors for statin-induced myopathy (older age, exercises, female sex) are not relevant to DMD boys. Nevertheless, due to the potential threat of muscle-related side effects, statins were overlooked in the past but it is probable that the work by Whitehead et al. [215] may start the new thinking about statins and muscle-related disorders (Fig. 6).

## Conclusions

Although a great amount of effort has been put into the investigation of novel therapies, there is still no effective cure for DMD patients, making this pathology one of the most dangerous and devastating disease affecting boys already in early childhood. Some new players and additional mechanisms contributing to DMD onset and progression have been identified recently. Among them, alterations in angiogenesis were reported both in animal models and DMD patients, therefore vascular-targeted therapy has been proposed as a treatment option for DMD to reduce ischemia and enhance endogenous repair processes. Nonetheless, more studies are warranted to fully understand the role of angiogenic mediators and disturbed blood vessels functioning in DMD pathology. It can be envisaged that the dystrophin deficiency in endothelial cells is either responsible or at least involved in the angiogenesis defects observed in DMD. On the other hand, it cannot be excluded that those alterations might be a consequence of systemic changes driven by the lack of dystrophin, extending far beyond the muscle weakening.
